# Spatial scales of genetic structure and gene flow in *Calochortus albus* (Liliaceae)

**DOI:** 10.1002/ece3.566

**Published:** 2013-04-15

**Authors:** Jillian M Henss, Jackson R Moeller, Terra J Theim, Thomas J Givnish

**Affiliations:** 1 Department of Entomology, University of Wisconsin-MadisonMadison, Wisconsin, 53706; 2 Department of Botany, University of Wisconsin-MadisonMadison, Wisconsin, 53706

**Keywords:** Endemism, parallel adaptive radiations, spatial genetic structure, species richness

## Abstract

*Calochortus* (Liliaceae) displays high species richness, restriction of many individual taxa to narrow ranges, geographic coherence of individual clades, and parallel adaptive radiations in different regions. Here we test the first part of a hypothesis that all of these patterns may reflect gene flow at small geographic scales. We use amplified fragment length polymorphism variation to quantify the geographic scales of spatial genetic structure and apparent gene flow in *Calochortus albus,* a widespread member of the genus, at Henry Coe State Park in the Coast Ranges south of San Francisco Bay. Analyses of 254 mapped individuals spaced 0.001–14.4 km apart show a highly significant decline in genetic identity with ln distance, implying a root-mean-square distance of gene flow σ of 5–43 m. STRUCTURE analysis implies the existence of 2–4 clusters over the study area, with frequent reversals among clusters over short distances (<200 m) and a relatively high frequency of admixture within individuals at most sampling sites. While the intensity of spatial genetic structure in *C. albus* is weak, as measured by the *Sp* statistic, that appears to reflect low genetic identity of adjacent plants, which might reflect repeated colonizations at small spatial scales or density-dependent mortality of individual genotypes by natural enemies. Small spatial scales of gene flow and spatial genetic structure should permit, under a variety of conditions, genetic differentiation within species at such scales, setting the stage ultimately for speciation and adaptive radiation as such scales as well.

## Introduction

*Calochortus* (Liliaceae *sensu* APG 2009) is a large genus (ca. 70 spp.) of bulbous geophytes, ranging from British Columbia east to the Dakotas and south to Guatemala, with a center of diversity in California (Ownbey 1940; Gerritsen and Parson 2007; Fiedler and Zebell 2012). The genus exhibits four distinct floral syndromes (mariposas, cat's ears, star tulips, fairy lanterns) and many species are visited by a wide range of pollinators, with limited divergence in the range of such pollinators visiting species in the same habitat (Jokerst 1981; Dilley et al. 2000) *Calochortus* occupies a wide range of habitats, including deserts, grasslands, chaparral, meadows, vernal pools, and forest and woodland understories, with most individual taxa restricted to narrow geographic areas (Ownbey 1940; Patterson and Givnish 2004; Fiedler and Zebell 2012). One in four species is restricted to specialized substrates, such as serpentine (Raven and Axelrod 1978; Kruckeberg 1986, 1992; Fiedler and Zebell 2012) or gypsum (Serna et al. 2005), and over 20% are considered federally endangered or extinct (Skinner and Pavlik 1994).

Patterson and Givnish (2004) used plastid sequence data to show that *Calochortus* includes seven major clades centered in different geographic regions; that individual floral syndromes, habitat preference, and serpentine tolerance have each evolved independently several times; and that closely related species are often nearest neighbors geographically. They proposed that narrow endemism, geographic coherence of individual clades, and parallel adaptive radiations in different areas might all have resulted from limited gene flow via seed dispersal over small spatial scales, resulting in spatial genetic structure (SGS) within species at small spatial scales, and in some cases ultimately resulting in speciation and endemism over limited scales as well (see also Givnish 2010; Claramunt et al. 2012). The biology of *Calochortus* suggests that at least one of the two components of gene flow in plants – seed movement – is likely to occur over small spatial scales. The seeds of *Calochortus* are relatively large, generally unwinged, and lack obvious adaptations for long-distance dispersal. Furthermore, Bullock (1976) found that the seeds of two *Calochortus* species (*C. catalinae, C. clavatus*) on an experimental slope after a heavy storm moved <2 m. In natural populations of *Calochortus westonii*, most seeds spill from the fruit within 15 cm of the parent plant, producing a tight clump of 20–30 seedlings the following spring (Knapp 1995). Knapp (1995) also found that seeds of *C. westonii* may be carried up to a few meters by water.

**Figure 1:**
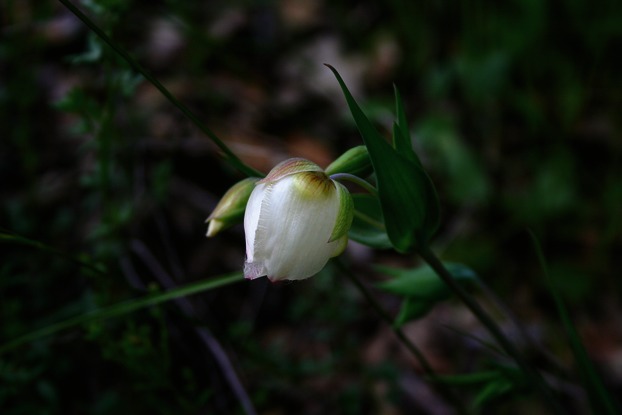
*Calochortus albus* in bloom at Henry Coe State Park (photo: Jillian Henss).

Here we use amplified fragment length polymorphism (AFLP) markers test the hypothesis that total gene flow in *Calochortus* occurs at small geographic scales, resulting in fine-grained SGS within species. AFLPs provide a powerful means for estimating genetic variation within and among plant populations (Vos et al. 1995), can produce large amounts of replicable data with a relatively small amount of effort compared with random amplified polymorphic DNAs or microsatellite DNA (Wolfe and Liston 1998; Mueller and Wolfenbarger 1999; Squirrell et al. 2003; Meudt and Clarke 2007), and have previously been used in several studies of gene flow and SGS in plants (e.g., Terro et al. 2003; Jacquemyn et al. 2004; Wilson et al. 2005; Szovenyi et al. 2009; Garrido et al. 2012). We employ AFLP genetic markers to quantify the relationship between genetic similarity and geographic distance in *Calochortus albus* (Fig. 1) over scales from 1 m to 15 km within the South Coast Ranges near San Francisco Bay, estimate the root-mean-square distance of total gene flow (σ), and calculate the strength of spatial genetic structure (*Sp*), and the size of its genetic neighborhoods (*N*_*b*_). This article is the first step of an investigation into genetic differentiation, introgressive gene flow, and phylogenetic relationships in the Bay Area clade of *Calochortus*.

## Methods

### Study material

*Calochortus albus* is herbaceous, with one ribbonlike basal leaf developing early each spring from a perennial bulb. Later in the spring, a single flowering stalk is produced, from which hang nodding, globe-shaped flowers (Fig. 1) that vary regionally in color from white to pale pink to red (Ownbey 1940). *C. albus* does not spread vegetatively. It is protandrous, with anthers of a given flower dehiscing before the stigma is receptive. Individuals are visited and pollinated exclusively by bees (mostly *Bombus*) and, although they are self-compatible, protandry, and pollinator observations by Jokerst (1981) suggest that selfing is rare.

*Calochortus albus* is a member of the Bay Area clade, a group of 10 species centered around San Francisco Bay, but ranging into more distant portions of the Coast Ranges, the Sierra Nevada, and the Cascades (Patterson and Givnish 2004). *C. albus* is the most widespread member of the Bay Area clade in California, and occurs in oak woodlands in the Coast Ranges south of San Francisco Bay, the foothills of the northern and central Sierras, the western Transverse Ranges, and the northern Peninsular Ranges.

### Sampling

**Figure 2:**
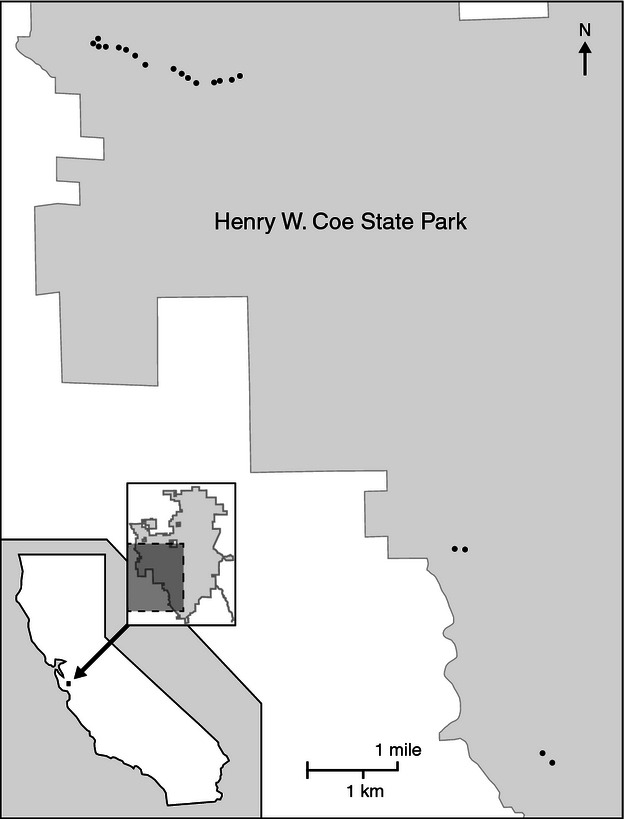
Map of the distribution of sample sites at Henry Coe State Park. There is an extensive, two-dimensional area of oak woodlands on north-facing slopes inhabited by *Calochortus albus* surrounding the essentially one-dimensional array of 16 sample sites in the northern portion of the park.

During spring 2006, 20 populations of *C. albus* were sampled at Henry Coe State Park (37°11′N, 122°33′W), a 350-km^2^ California state park located southeast of San Jose in the interior South Coast Ranges (Fig. 2). Sixteen sample sites were located in oak woodlands on north-facing slopes throughout the western section of the park along Flat Frog Trail, Forest Trail, and Poverty Flat Road; four other sites were located in the southern section, along Grizzly Gulch Trail and Hunting Hollow Road. Although the 16 northern sites form a nearly one-dimensional array (Fig. 2), oak-woodland habitat for *C. albus* is strongly two-dimensional there, covering large areas of the north-facing slopes. Distances between populations ranged from 0.07 to 14.4 km, and distances between plants within populations varied from 0.2 to ca. 50 m. Within this area, *C. albus* was extensively distributed and especially common on north-facing slopes. In each population, leaf material was collected from 10 to 20 individuals and preserved in silica gel, sampling a total of 254 plants. Universal Transverse Mercator coordinates of one individual per population were determined using a high-precision GPS (Leica SR530, St. Gallen, Switzerland; 1 cm horizontal precision). Coordinates of all other individuals were determined based on their bearing and distance from the focal plant using a compass and a sonic range-finder (Sonic Multi-Measure™ ComboPro #10300, Charlotte, NC; 10 cm horizontal precision). In each study population, and at 10 sites randomly located between populations, reproductive and nonreproductive individuals were counted along three 1 m × 10 m transects, to produce estimates of the density of reproductives and nonreproductives.

### AFLP data generation

High-quality genomic DNA was extracted using a DNeasy® 96-well plant extraction kit (Qiagen, Valencia, CA). AFLP data were generated following the protocols of Myburg et al. (2001), with minor modifications for optimization with an ABI 3100 capillary sequencer (Applied Biosystems, Foster City, CA). Genomic DNA samples were digested with the restriction endonucleases *Eco*RI and *Mse*I in a 10 μL reaction containing 83 ng DNA, 0.05 μL 100 ng/100 μL BSA, 5 U *Eco*RI, and 5 U *Mse*I and incubated at 37°C for 2 h. Double-stranded adapters were then ligated to the resulting digestion fragments. Double-stranded adapters were produced by mixing equal volumes of equimolar solutions of two oligos for both the M (5′ GAC GAT GAG TCC TGA G 3′ and 5′ TAC TCA GGA CTC AT 3′) and the E (5′ CTC GTA GAC TGC GTA CC 3′ and 5′ ATT TGG TAC GCA GTC TAC 3′) adapters. These solutions were then incubated at 95°C for 5 min and allowed to cool 1°C per minute at room temperature. A volume of 0.19 μL of both the M and the E adapters, along with 3.52 μL ddH_2_O, 1 μL 10 × ligase buffer, and 0.4 U T4 DNA ligase were added to the 5 μL digestion reaction and incubated at 16°C overnight. Ligation products were then diluted at a ratio of 17 μL ligation product to 70 μL ddH_2_0.

Pre-selective amplification reactions were carried out using the dilute ligation products and primers complementary to the adapters but extending one additional, specified base in the 3′ direction. These 25 μL reactions contained 2.5 μL 10 × buffer, 1.5 μL 25 mmol/L MgCl_2_, 2 μL 2.5 mmol/L (each) dNTPs, 0.38 μL M+C primer, 0.38 μL E+A primer, 1.5 U *Taq*, 5 μL dilute ligation product, and 13 μL ddH_2_0. Reactions were then cycled under the following conditions: 72°C for 60 sec; 20 cycles of 94°C for 50 sec, 56°C for 60 sec, 72°C for 120 sec; 72°C for 120 sec. Pre-selective amplification products were then diluted with ddH_2_O at a ratio of 40 μL: 720 μL.

Three rounds of selective amplifications were performed using dilute pre-selective amplification products and three different primer pair combinations. All of the primers used in this final selective amplification were complementary to those used in the pre-selective amplification but extended an additional two or three bases in the 3′ direction. The primer pair combinations used were as follows: M+CCAG and E+ATT, M+CTT and E+ACT, and M+CCCG and E+AGC. In each primer pair the E+A– primer was fluorescently labeled. These 25 μL reactions contained 2.5 μL 10 × buffer, 1.5 μL 25 mmol/L MgCl_2_, 3 μL 2.5 mmol/L (each) dNTPs, 0.5 μL deionized formamide, 1.25 μL M+C— primer, and 0.25 μL labeled E+A– primer, 1.25 U *Taq*, and 10.75 μL ddH_2_O. The reactions were then exposed to the following cycling conditions: 10 cycles of 94°C for 50 sec, 65°C for 60 sec (decreasing by 1°C each cycle), 72°C for 120 sec; then 20 cycles of 94°C for 50 sec, 56°C for 60 sec, 72°C for 120 sec; then 72°C for 10 min.

Selective amplification products were cleaned using magnetic beads (CleanSeq™, Agencourt, Beverly, MA) and run on an ABI 3100 capillary sequencer using a fluorescent internal standard in each lane (Geneflow™ 625, Chimerx, Milwaukee, WI). Chromatograms were analyzed using GeneMarker (SoftGenetics LLC, State College, PA) to generate 0/1 matrices of fragments 100–300 bp in length for the M+CCAG/E+ATT and M+CCCG/E+AGC primer pair combinations and 100–400 bp in length for the M+CTT/E+ACT primer pair combination.

### Analysis of spatial genetic structure

Individual AFLP bands were each assumed to represent one locus with two alleles. The presence of a band thus indicated either a heterozygote or dominant homozygote at that locus, while the absence of a band indicated a recessive homozygote. Spatial genetic structure was assessed by calculating the slope of pairwise kinship coefficients (Hardy 2003) against the logarithm of distance between individuals, using the software program *SPAGeDi* 1.3 (Hardy and Vekemans 2009). The kinship coefficient was developed for dominant genetic markers and thus requires an estimate of the inbreeding coefficient, but is robust to moderate errors in that coefficient (Hardy 2003). Given the strong protandry seen in *C. albus,* we conducted calculations assuming Hardy–Weinberg conditions and an inbreeding coefficient of zero. Pairs of samples were binned into nine, logarithmically spaced distance classes: 0–3 m, 3–9 m, 9–27 m, 27–81 m, 81–243 m, 243–729 m, 729–2187 m, 2187–6561 m, and 6561–19683 m. For each of these classes, average pairwise kinship values were plotted against ln distance to create a kinship-distance plot (Hardy 2003). Least mean squares regressions were used to determine the slope of the regression in the kinship-distance plot using average values for distance classes across all 20 sites, and for the 16 northern sites only. For all pairwise comparisons of individual plants, Mantel tests based on 999 permuations of the data were used to determine whether regression slopes differed significantly from zero for plants from all 20 sites, and for those from the 16 northern sites only (Hardy 2003).

To permit comparisons with results from other studies and to estimate neighborhood size (*N*_*b*_), we calculated the *Sp* statistic (Vekemans and Hardy 2004). *Sp* is a measure of the strength of SGS, with high values indicating strong fine-scale structure. *Sp* is defined as


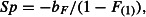
(1)

where b_F_ = the slope of the regression of kinship on ln geographic distance and F_(1)_ = the average kinship between adjacent plants. The average kinship between plants falling into the first distance category (0–3 m) was used to estimate F_(1)_. The *Sp* statistic was then used to estimate the root-mean-square distance of gene dispersal (σ) as


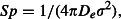
(2)

where *D*_*e*_ is effective population density and σ is the root-mean-square distance of gene dispersal (Vekemans and Hardy 2004). Neighborhood size (*N*_*b*_) (Wright 1943, 1969) was calculated as the inverse of *Sp* (Vekemans and Hardy 2004). Estimates of σ and *N*_*b*_ were made using an iterative procedure to estimate σ based on the genetic structure over a restricted distance range. Equations (1) and (2) hold best over distances between σ and 20σ (Vekemans and Hardy 2004). Therefore, *SPAGeDi* applies an iterative regression procedure within this range, first calculating an *Sp* value from the slope of the regression of the kinship coefficient on ln distance over an arbitrarily chosen initial range of distances, and then using this *Sp* value to calculate σ according to equation (2). A restricted regression is then calculated over distances between σ and 20σ, and a new *Sp* value obtained based on the slope over this range. This procedure is repeated 100 times or until estimates of σ converge on a stable value (Hardy and Vekemans 2009), thus providing an estimate of the scale of gene dispersal at a given effective density as well as *N*_*b*_. We confirmed, in each case, that the same estimates of σ, *Sp*, and *N*_*b*_ resulted when the iterative procedure was started using interplant distances of 10–200 m as when starting at interplant distances of 100–2000 m.

We calculated the mean ± SD of the densities of reproductive and nonreproductive individuals across all 20 sites surveyed. We estimated effective population density *D*_*e*_ as the density *D* of reproductive individuals times 0.5, 0.3, and 0.1, given that effective densities of natural plant populations often fall within this range (Husband and Barrett 1992; De-Lucas et al. 2009). We estimated σ, *Sp*, and *N*_*b*_ for a total of nine estimates of *D*_*e*_, based on the mean density *D* of reproductives observed, plus or minus one standard deviation, multiplied by the factors 0.5, 0.3, or 0.1.

We compared the values of the *Sp* statistic for *C. albus* with those of other herbaceous plants in the meta-analysis of Vekemans and Hardy (2004), to determine whether *C. albus* showed exceptionally short dispersal distances. Comparisons included the placement of taxa into one of four categories based on pollination mechanism and mode of seed dispersal: (1) animal pollination/gravity dispersal; (2) animal pollination/animal dispersal; (3) wind pollination/gravity dispersal; and (d) animal pollination/mixed animal and gravity dispersal. The latter permitted us to assess whether *C. albus* had exceptionally short dispersal distances given its ecology of pollen and seed dispersal.

### Cluster analyses

We employed the Bayesian clustering algorithms in STRUCTURE v. 2.3.4 (Pritchard et al. 2000; Falush et al. 2003; Hubisz et al. 2009) to infer population structure and to assign individuals to clusters, based on multi-locus genetic data and minimization of Hardy–Weinberg disequilibrium within clusters. The estimation analyses assume different numbers of clusters *K*, and then compare the estimated log probability of data under each *K*, ln Pr(X|*K*). We conducted 20 replicate runs for all proposed values of *K* between 1 and 10, assuming dominant AFLP markers, admixture among clusters and individuals (α = 1), default allele frequency distribution (λ = 1), and correlated allele frequencies. Each run used 10^4^ iterations following a burnin period of 5 × 10^4^ iterations. We estimated the number of clusters as the value of K with the greatest Pr(X|*K*), and then tested that using the Δ*K* procedure of Evanno et al. (2005). We compiled color-coded STRUCTURE plots of plant membership in individual cluster(s) to assess spatial population structure, plotting sample sites in order from west to east. We conducted a parallel set of analyses restricting attention solely to the northern 16 sample sites, excluding the large distances to the two pairs of southern sample sites (Fig. 2).

## Results

### AFLPs

For the 254 individuals sampled, the primer pair M+CCAG/E+ATT generated 136 scoreable AFLP loci, with 132 of these being variable. The primer pair M+CTT and E+ACT generated 206 scoreable loci, 199 of which were variable; the primer pair M+CCCG and E+AGC generated 142 scoreable loci, of which 140 were variable. AFLPs for all primer pairs thus provided 471 variable loci out of 484 (97.3%) loci scored.

The number of pairs of individuals at various distances ranged from 96 in the smallest distance class (<3 m) to 12,160 in the largest distance class (>6561 m) (Table 1). The percentage of bands participating in each class ranged from 50.8% in the <3 m class to 100% participation in the >6561 m distance class; percent participation was >50% for all other classes (Table 1).

**Table 1:** Description of upper and lower bounds, mean inter-individual distance, number of pairs, and psercentage participation for each distance class

Distance class	1	2	3	4	5	6	7	8	9
Max distance (m)	3	9	27	81	243	729	2187	6561	19683
Min distance	0.21	3.01	9.01	27.01	81.01	243.01	729.01	2187.01	6561.01
Mean distance	1.96	6.21	15.78	51.23	179.04	500.39	1396.20	2702.95	11747.68
# of pairs	96	444	942	318	2169	4272	8711	3019	12160
% participation	50.8	90.2	99.2	57.1	87.0	87.8	74.8	64.2	100.0

Percentage participation indicates the fraction of all individuals that participated in at least one pairwise comparison within the distance class in question.

### Spatial genetic structure

**Figure 3:**
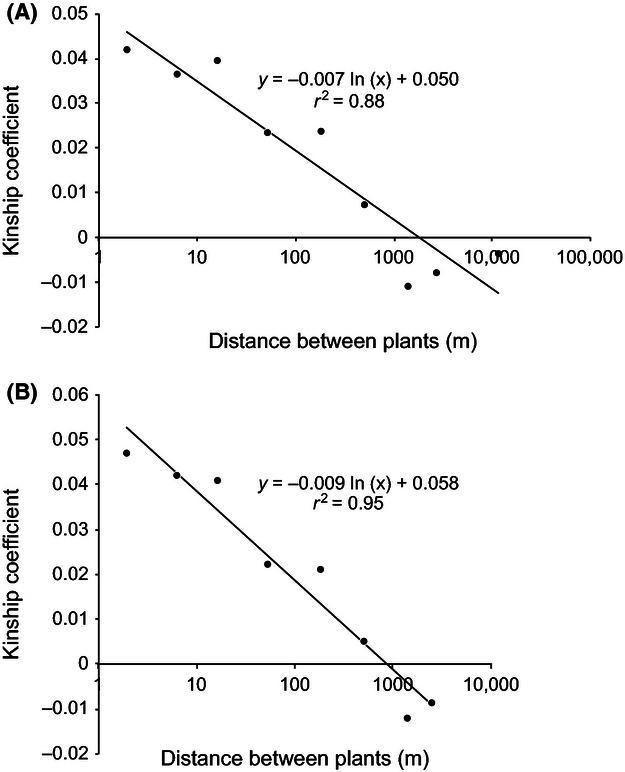
Mean ± SD kinship coefficient plotted against the logarithm of geographic distance for each distance class. (A) All 20 sites; (B) the northern 16 sites only).

Kinship values, averaged over each distance class, declined in a highly significant fashion with the logarithm of distance between individuals, for all sites (y = −0.0067 ln x + 0.050, *r*^*2*^ = 0.883, *P* < 0.002 for two-tailed *t*-test with 7 df) or for just the 16 northern sites (y = −0.0086 ln x + 0.058, *r*^*2*^ = 0.946, *P* < 0.0004 for 6 df) (Fig. 3A and B). Over all individual pairs of individuals, kinship values declined slightly less sharply with ln distance, but with less than 5% of the explanatory value, for all sites (y = −0.0051 ln x + 0.038, *r*^*2*^ = 0.044) or for just the 16 northern sites (y = −0.000016 ln x + 0.017, *r*^*2*^ = 0.070); both patterns were significant under Mantel tests.

The density *D* of reproductive *C. albus* was 0.50 ± 0.33 individuals m^*−2*^; that of nonreproductives, 1.57 ± 1.47 individuals m^*−*2^. Assuming that *D* = 0.16, 0.50, and 0.83 individuals m^*−*2^, and that *D*_*e*_ = 0.5*D*, 0.3*D*, and 0.1*D*, we estimated that the root-mean-square distance of gene dispersal σ was 16.7 ± 10.6 m, averaged across the study region, with a range of 5.2–42.9 m (Table 2). For the northern 16 populations, the comparable estimates were σ = 15.4 ± 11.5 m, with a range of 4.7–42.5 m (Table 3). Overall, half of the iterative calculations of σ converged on a single value, while the others converged on a cycle within a narrow range (Tables 2 and 3). Generally, estimates of σ decreased with plant density and the estimated fraction g of reproductive plants. Across the nine combination of plant density and g, estimates of σ based on the 16 northern sample sites and all 20 sites were tightly coupled (*r* = 0.98, *P* < 0.0001). For all sample sites, we estimated that neighborhood size *N*_*b*_ was 140 ± 47 individuals, with a range of 69–193; the *Sp* statistic was 0.00404 ± 0.00157, with a range from 0.00259 to 0.00722 (Table 2). For calculations restricted to the northern 16 sample sites, *N*_*b*_ = 125 ± 53 (range: 52–194) and *Sp* = 0.00575± 0.00277 (range: 0.00275–0.00967) (Table 3).

**Table 2:** Estimated values of σ, *N*_*b*_, and *Sp* for different combinations of reproductive density *D* and the fraction *g* of *D* yielding effective population size (*D*_*e*_ = *gD*) for all 20 populations

		*D* = 0.16 m^−2^	*D* = 0.50 m^−2^	*D* = 0.83 m^−2^
*g* = 0.5	σ	15.6 (13.1–18.1)	11.1	5.2
	*N*_*b*_	125.5	192.8	69.2
	*Sp*	0.00408	0.00259	0.00722
*g* = 0.3	σ	17.8 (16.1–19.6)	13.4	11.1
	*N*_*b*_	96.6	169.8	193.1
	*Sp*	*0*.00522	0.00294	0.00259
*g* = 0.1	σ	42.9	17.8 (15.8–20.4)	15.4 (13.2–17.7)
	*N*_*b*_	185.3	101.1	127.1
	*Sp*	0.00270	0.00501	0.00402

**Table 3:** Estimated values of σ, *N*_*b*_, and *Sp* for different combinations of reproductive density *D* and the fraction *g* of *D* yielding effective population size (*D*_*e*_ = *gD*) for the northern 16 populations

		*D* = 0.16 m^−2^	*D* = 0.50 m^−2^	*D* = 0.83 m^−2^
*g* = 0.5	σ	15.7 (13.9–17.5)	5.7	4.7
	*N*_*b*_	125.6	51.7	58.2
	*Sp*	0.00403	0.00967	0.00860
*g* = 0.3	σ	18.1 (16.0–20.2)	12.5	5.7
	*N*_*b*_	96.6	169.8	193.1
	*Sp*	0.00505	0.00338	0.00967
*g* = 0.1	σ	42.5 (29.6–57.6)	18.3 (15.4–21.8)	15.7 (14.0–17.3)
	*N*_*b*_	194.1	107.3	129.8
	*Sp*	0.00275	0.00475	0.00388

The average estimate of *Sp* = 0.00404 for *C. albus* is the lowest for any herbaceous species tabulated by Vekemans and Hardy (2004), although the upper limit of the range of values calculated exceeds the observed *Sp* for several animal pollinated, gravity (or wind) dispersed herbs. The latter category includes 17 of the 24 herbs tabulated by Vekemans and Hardy (2004), and by far the lowest minimum values of *Sp*. This indicates that *C. albus* has a relatively weak pattern of SGS. However, the estimated spatial scale of gene flow σ = 16.7 m (range up to 42.9 m) is strikingly short in absolute terms, providing *prima facie* evidence of gene flow restricted to small spatial scales.

### Cluster analyses

**Figure 4:**
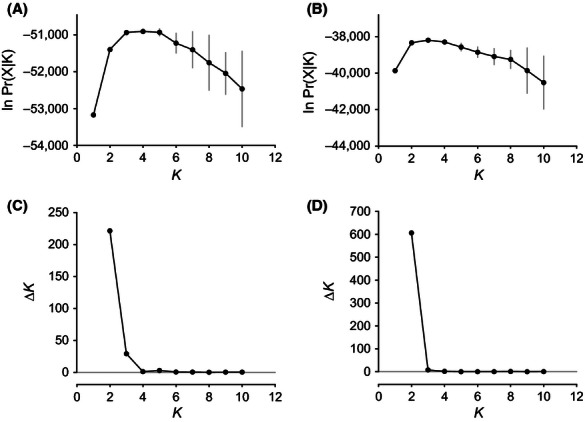
Plot of Pr (X|*K*) against *K*, based on data for (A) for all 20 sites, and (C) the 16 northern sites. Error bars represent standard deviations after the burn-in period. The plateau of these curves suggest a *K* between 2 and 5. Δ*K* versus *K*, implying a *K* = 2 for (B) all 20 sites and (D) the 16 northern sites.

**Figure 5:**
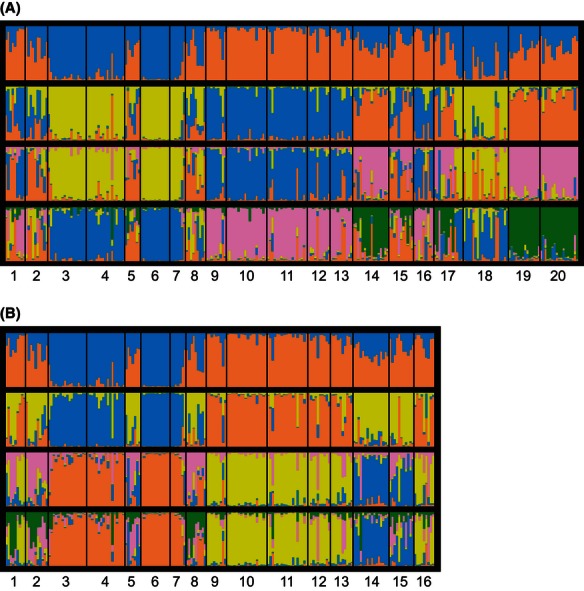
(A) STRUCTURE plots of individual membership in two, three, four, or five color-coded clusters for all 20 sites. Black vertical lines separate sets of individuals at different sites, which are ordered from left to right in their geographic sequence from west to east at Henry Coe State Park. Admixture of indivuals is indicated by the presence of two or more cluster colors within individual vertical bands. (B) Plots of individual members in two, three, four, or five clusters for the northern 16 sites.

STRUCTURE identified 2–4 clusters across either the 16 northern sample sites or all 20 sites, based on a plateauing of the Pr (X|*K*) curves between *K* = 2 and *K* = 5 (Fig. 4A and C). The ΔK procedure strongly suggested an optimal number of clusters at *K* = 2 in both cases (Fig. 4B and D). STRUCTURE showed very small standard deviations of Pr (X|*K*) after the burnin period for all but the largest number of implied clusters. Plots of cluster membership against the spatial order of the sample sites for *K* = 2 to *K* = 5 indicated a repeated set of rather steep transitions from populations dominated by one cluster to the other, on the scale of tens of meters, and a substantial fraction of admixed individuals at several sample sites (Fig. 5A and B). Thus, cluster analysis appears to be quantitatively consistent with our analyses of spatial genetic structure and the spatial scale of gene flow, and suggests repeated dispersal events by members of different clusters, followed by proliferation of their progeny.

## Discussion

The data presented in this article support the hypothesis of Patterson and Givnish (2004) that seed dispersal and overall gene flow (including pollen) in *Calochortus* occurs over relatively small distances. Our data on SGS imply that the root-mean-square distance of gene dispersal σ is between 5 and 43 m, and that neighborhood size is between 52 and 194 individuals. Limited gene flow provides a potential explanation for local differentiation seen within species of *Calochortus* (e.g., in *C. albus* from the northern Sierras vs. south Coast Ranges [Ownbey 1940]), the high level of local endemism seen across species, the geographic coherence of individual clades of *Calochortus*, and the parallel adaptive radiations the genus has undergone for several traits in different areas. Further study is needed, however, to determine whether such limited gene flow is characteristic of *Calochortus* as a whole, and the extent to which it reflects variation in seed versus pollen dispersal distance.

AFLP markers are not ideal for estimating the spatial scale of gene flow. In particular, their dominant nature makes it impossible to separate heterozygotes from dominant homozygotes, and their essentially uniform origin in the biparentally inherited nuclear genome makes it impossible to separate gene flow via pollen from that via seeds. Ideally, use of co-dominant markers (e.g., microsatellite DNAs or RAD-Seq markers) coupled with a two-generation analysis of adults and maternal and biparental tissue in dispersed seedlings produce the most powerful estimates of both pollen and seed dispersal (e.g., see Smouse et al. 2001, 2012; Sork et al. 2002). However, such an approach did not prove workable for *Calochortus*. Our aggregate estimate of gene flow in *Calochortus* based on AFLP variation almost surely overestimates the spatial scale of seed movement, which Patterson and Givnish (2004) argued was the basis for frequent speciation, geographic cohesion, and parallel patterns of adaptive radiation in *Calochortus*. Our estimates of σ are thus conservative over-estimates of seed dispersal, and support a critical assumption of the Patterson–Givnish hypothesis.

The slope *Sp* of the regression of pairwise kinship values against ln distances provides a measure of the *intensity* of fine-scale SGS that is independent of the artificial delimitations of individual populations and directly captures the magnitude of genetic differences caused by isolation by distance (Vekemans and Hardy 2004). While *C. albus* is remarkable for the small scale at which gene flow is inferred to incur, it also shows an apparently very low intensity of genetic differentiation, with its value of *Sp* being smaller than most other herbs tabulated by Vekemans and Hardy (2004).

One potential shortcoming of the *Sp* statistic, however, is that its calculation almost insures that its value will be low in species that show low absolute levels of local kinship. That is, the slope of kinship against ln distance must be low if the maximum values of kinship itself are small. The adjustment of this slope for the kinship of nearby plants (see eq. (1)) increases rather than decreases this bias. *C. albus* has unusually low values of average kinship (ca. 0.04 within sites, all <100 m across), which may cause it to appear to have less intense genetic structure than other taxa (e.g., *Psychotria*, Theim 2006) with higher levels of local kinship. The causes of low local kinship in *C. albus* are unclear at this time, but might include features of the species that promote exceptionally high levels of local genetic diversity, such as repeated recolonizations of individual patches by seeds produced from a variety of other sites (Jones et al. 2006), or pathogen-driven density-dependent mortality of different genotypes (Rouse et al. 2011). Repeated transitions among clusters over short distances and relatively high proportions of admixed individuals in several sample sites in the STRUCTURE analysis are consistent with either of these possibiities. The small geographic scale at which gene flow does occur in *C. albus* should facilitate, at least under certain circumstances – such as occasional long-distance seed dispersal, or strong local selection for single genotypes – local differentiation within species which, ultimately, might lead to speciation and endemism at limited spatial scales. Given the very short, average distance of dispersal in *C. albus*, rare episodes of long-distance dispersal – perhaps via wind dispersal associated with strong storms – would almost certainly have been needed for this species to have succeeded in colonizing its present extensive range at mid elevations on either side of California's Central Valley. Occasional dispersal events, or range shifts resulting from climatic change, should leave traces in spatial genetic structure at large scales within species or species complexes (Petit et al. 1997; Dick et al. 2003; Dutech et al. 2003; Chung et al. 2004; Dick and Heuertz 2008; Zhao et al. 2011). The short distances over which gene flow and genetic differentiation occur in the common species *C. albus* should be carefully considered in attempts to conserve or restore populations in other, rarer members of the same genus.
